# Prediction of total digestible nutrient and crude protein requirements according to daily weight gain, and behavioral measurements of Hanwoo heifers

**DOI:** 10.5713/ab.22.0387

**Published:** 2023-01-11

**Authors:** Ju Ri Kim, Jun Sik Woo, Youl Chang Baek, Sun Sik Jang, Keun Kyu Park

**Affiliations:** 1 Department of Animal Science and Technology, Konkuk University, Seoul 05029, Korea; 2 National Institute of Animal Science, Rural Development Administration, Jeonbuk 55365, Korea

**Keywords:** Animal Behaviors, Hanwoo, Heifers, Nutrients, Requirements

## Abstract

**Objective:**

This study was conducted to investigate the effects of energy and protein levels in the diet of Hanwoo heifers on growth response and animal behavior.

**Methods:**

Forty heifers were randomly allocated into three experimental groups according to the target daily weight gain in 8 pens (T-0.2, 2 replications; T-0.4 and −0.6, 3 replications) based on similar body weight (BW) and age in months. The target average daily gain (ADG) was set at 0.2 (T-0.2), 0.4 (T-0.4), and 0.6 kg/d (T-0.6), and feed was based on National Institute of Animal Science (NIAS, 2017). In order to minimize hunger stress of T-0.2 and −0.4, the feeding ratio of rice straw was set to 55%, 50%, and 45% for T-0.2, −0.4 and T-0.6, respectively, so that the dry matter (DM) intake for all treatment groups was uniform but the energy and protein levels in the diet were adjusted differently. A total of 6 items (lying, standing, eating, rumination, walking and drinking) of animal behavior were analyzed.

**Results:**

During the whole period of the experiment, the ADG of the T-0.2, −0.4 and −0.6 treatments were 0.48, 0.56, and 0.65 kg/d (p<0.05), respectively, showing higher gain than the predicted value, especially for the low target ADG group. Based on these results, regression equations for the total digestible nutrient (TDN) and crude protein (CP) requirements were derived. No behavioral differences were found according to the energy and protein levels in the diet because the DM intake was kept constant by adjusting the roughage and concentration ratio. However, eating time was longer (p<0.05) at T-0.2 than T-0.6 during the whole day.

**Conclusion:**

Through this study, it was possible to derive regression equations for predicting TDN and CP requirements according to the target ADG and BW.

## INTRODUCTION

The purpose of raising economic animals is to improve animal productivity and economy of livestock farm through optimal nutritional feeding. If nutrient requirements are accurately predicted and supplied to livestock, useful livestock products can be produced more efficiently and waste of valuable feed resources can be prevented. For this purpose, many countries establish their own livestock feeding standards based on nutrient requirements using actual measurements obtained through various studies and provide guidelines for its use in livestock farms. In Korea, the feeding standard for Hanwoo (*Bos taurus coreanae*), Korean native beef cattle, has been revised every five years since 2002. However, although many studies have been conducted on the nutrient requirements of steers and the improvement of meat quality by increasing marbling, studies on the exact nutrient requirements of Hanwoo cows are extremely lacking [1,2].

The growing period of cows is a period of maximum growth of skeletons, muscles, in ternal organs and reproductive organs, thus being the most important period because it determines the overall productivity of a cow. The first onset of estrus of heifers is mainly affected by weight rather than the age of the individual, and most of the *B. taurus* varieties such as Angus, Charolais, Hereford, etc. to which Korean beef cattle belongs are considered to reach puberty at approximately 60% of the mature weight [3]. Therefore, in order to maximize the health and reproductive efficiency of heifers, it is necessary to induce appropriate growth by supplying nutrients according to the exact requirements. Feeding nutrients higher than the required amount causes excessive growth, which negatively affects milk production [4] and mammary gland development [5]. On the other hand, insufficient nutrient feeding slows the growth of individuals and delays estrus and the first pregnancy, thereby lowering the productivity of livestock [6]. This study was conducted to examine the growth response and animal behavior of Hanwoo heifers according to the level of energy and protein intake to derive the appropriate nutrient supply level.

## MATERIALS AND METHODS

### Animal care

The animal use and protocols employed during the research were reviewed and approved by the Institutional Animal Care and Use Committee at Konkuk University (Approval number: KU20112).

### Chemical analysis

All samples of diets were dried at a forced-air oven for over 24 hours at 105°C and ground through 2 mm screen using a Wiley mill (Model 4; Thomas Scientific, Swedesboro, NJ, USA). Samples were analyzed for dry matter (DM) (AOAC official method 930.15) [7], crude protein (CP) (AOAC official methods 984.13), ether extract (EE) (AOAC official methods 2003.05), crude fiber (AOAC official method 942.09), ash (AOAC official method 942.05), neutral detergent-insoluble fiber (NDF) [8], acid detergent-insoluble fiber (ADF), and lignin [9].

### Animal trial

Forty heifers (16.7±1.3 months age, initial body weight [BW] 318.5±27.9 kg) were randomly allocated into three experimental groups according to the target daily weight gain, consisting of five animals per pen in a total of 8 pens (T-0.2, 2 pens; T-0.4, and −0.6, 3 pens) based on similar BW and age in months. The experiment was conducted for a total of 63 days. The appropriate average daily gain (ADG) for heifers suggested by National Institute of Animal Science (NIAS; 2012, 2017) [1,2] is 0.55 kg/d. Therefore, the target ADG in this experiment was set at 0.2, 0.4, and 0.6 kg/d, respectively. Feeds were offered according to the target ADG based on NIAS (2017) [2], and there was no adaptation period because the animals were fed the same mixed concentrates (formula feed) and roughage (rice straw) that were previously consumed. In order to minimize hunger stress of T-0.2 and −0.4, the feeding ratio of rice straw was set to 55%, 50%, and 45%, respectively, so that there was no difference in DM intake (DMI) among treatment groups, but the energy and protein levels in the feed became different. Feeds were offered twice equally at 07:00 and 17:00 h daily. Animals could access fresh water and mineral block without any restriction during the whole period. The nutrient content of the feed used in the experiment and the mixing ratio of the formulated feed are shown in Table 1. The BW was measured at the initial stage of the experiment, the second (36 d), and the end of the experiment (63 d). The feed intake was measured every week for calculating feed conversion ratio (FCR; feed/gain).

### Observation of animal behaviors

Animal behavior was observed through CCTV (HIKVISION color camera, DS-2CE16D0T-IRP 3.6 mm) recorded for 24 hours consecutively for 3 days by selecting 6 heads per treatment group. The selected individuals were marked with fluorescent paint on their backs so that they could be identified by CCTV. Two CCTVs were installed diagonally in the front and back of each pen so that the entire pen could be observed without a blind spot.

A total of six observation items are standing, walking, eating, drinking, rumination and lying, and the observation criteria are shown in Table 2. The rumination behavior includes both lying and standing states, and standing includes walking, eating, rumination and drinking. Based on the sunrise and sunset times at the time of the experiment, observations were divided into day time (0700 to 1800, 11 h) and night time (1800 to 0700, 13 h) [10].

### Calculations and statistical analysis

Non-fiber carbohydrate (NFC), total digestible nutrient (TDN), and FCR values of experimental feeds were calculated as follows: i) NFC calculated by difference: 100−[CP+ (NDF−NDICP)+EE+ash]. ii) TDN = 0.93×CP+0.92×(1+EE−ash−CP−NDF)+0.75×(NDF−ADL)×(1−ADL^2/3^/[NDF]^2/3^) [11]. iii) FCR = DMI (kg)/gain (kg). Where, NDICP, neutral detergent insoluble CP; ADL, acid detergent lignin.

Experimental data were analyzed using the MIXED pro cedure of SAS (SAS Inst. Inc., Cary, NC, USA). The statistical model included the three treatments as a fixed variable and the animals as a random variable. Least squares means among treatments were compared using the PDIFF option when the treatment effect was significant. Statistical significance was determined at a p-value less than 0.05.

## RESULTS AND DISCUSSION

### Animal performance

The BW, DMI, ADG, and FCR of treatments according to the energy and protein levels in the feed of Hanwoo heifers are presented in Table 3. Initial (0 day) BW of T-0.2, −0.4, and −0.6 were 316.7, 318.3, and 320.6 kg, respectively. There was no significant difference in BW among treatments during the whole experimental period. However, T-0.2, −0.4, and −0.6 in final BW (63 day) were 348.3, 353.6, and 361.2 kg, respectively, and the weight gain difference during the whole period between T-0.2 and −0.6 was 9.0 kg.

The DMI of T-0.2, −0.4, and −0.6 was 5.75, 5.88, and 6.07 kg/d, respectively, and a constant amount was ingested with almost no residual throughout the entire period of the experiment. Thus, there was no significant difference among treatment groups. This is because the level of energy and protein required for weight gain by treatment groups was adjusted by setting different ratios of rice straw and formula feed, not DMI. In other words, the feeding ratios of rice straw for T-0.2, −0.4, and −0.6 were 55% (3.07 vs 2.67 kg for rice straw and formula feed), 45% (2.85 vs 3.04 kg) and 40% (2.63 vs 3.44 kg), respectively. Accordingly, the TDN intakes of T-0.2, −0.4, and −0.6 were 2.58, 2.75, and 2.95 kg/d, and the CP intakes were 0.54, 0.59, and 0.64 kg/d, respectively.

In the 1st period, the ADG of T-0.2, −0.4, and −0.6 was 0.59, 0.57, and 0.62 kg/d, respectively, and there was no significant difference. This is probably because the nutritional status of heifers before the experiment period was relatively good (ADG about 0.62), so even if the nutritional level was lower than the required amount for T-0.2 and −0.4, the difference between the treatment groups did not appear immediately after one month. The ADG in the 2nd period was 0.36, 0.56, and 0.72 kg/d, respectively, and showed a significant difference (p<0.05) among treatments. However, the ADG values of the T-0.2 and −0.6 treatment groups differed only twice, not three times. Although there was no significant difference in the first period, the result from the 1st period was not excluded from the results of the entire period because the weight appears as an accumulated result. When combined with those of the 2nd period, the ADG of the whole period was 0.48, 0.56, and 0.65 kg/d (p<0.05), respectively. The target ADG (T-0.2, 0.4, and 0.6 kg/d) was set based on NIAS (2017) [2], but the experimental results showed higher values than the target ADG in all treatment groups. Based on the results of this experiment, the ADG presented by NIAS (2017) [2] is probably underestimated, and more experiments on the energy and CP requirements of Hanwoo heifers are deemed necessary in the future.

**Figure 1:**
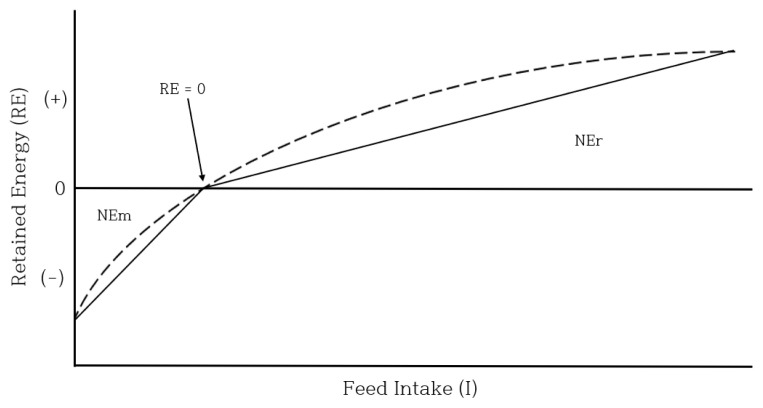
Relationship between RE (retained energy) and NE (net energy). The dashed line shows the curvilinearity and the solid lines are linear approximations (Adopted from [3]).

In addition, the lower the target daily weight gain, the greater the difference from the predicted weight gain with a relatively higher weight gain, i.e. T-0.2 (target ADG) vs 0.48 (result). This is probably because the regression equation of nutrient requirement is linear. In general, net energy (NE) for maintenance and weight gain is measured through the ratio of the changes in energy used for accumulation in the animal body (retained energy, RE) and energy consumed through feed (intake energy, IE); NE = ΔRE/ΔIE. The measurement of NE using this method assumes that the relationship between ingested feed and stored energy is linear. However, in the actual animal body, it has a curved reaction rather than a straight line, and the rate of energy stored decreases as feed intake increases (Figure 1) [3]. Thus, the higher the weight gain, the lower the efficiency of use of the ingested feed. Conversely, the lower the weight gain, the higher the feed utilization efficiency. The curvilinear relationship between ingested feed and stored energy consists of two aspects: i) ingested feed and reduced body tissue (− RE) and ii) ingested feed and increased body tissue (+ RE). Comparing the slopes between − and + RE, it can be seen that the feed consumed for net energy for maintenance (NEm) is more efficiently used than for net energy retained (NEr). Therefore, while the animal’s response is curved, the energy requirement is determined through a single linear regression equation. Accordingly, it is interpreted that the treatment group with a relatively low ADG showed a higher weight gain.

**Figure 2:**
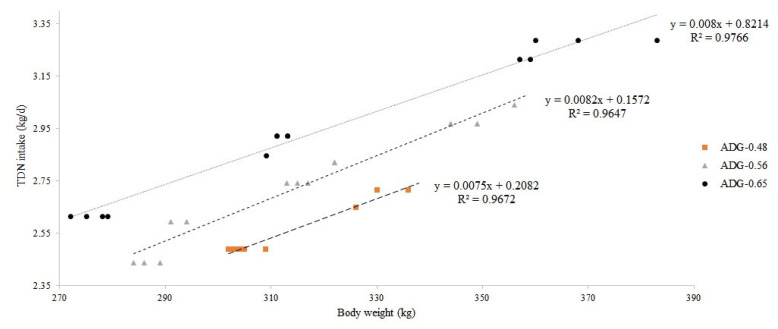
Regression equation for calculation of total digestible nutrient (TDN) requirements (kg/d) of Hanwoo heifers. ADG, average daily gain. ADG-0.48, y = 0.0075x+0.2082 (R^2^ = 0.9672); ADG-0.56, y = 0.0082x+0.1572 (R^2^ = 0.9647); ADG-0.65, y = 0.0080x+0.8214 (R^2^ = 0.9766).

**Figure 3:**
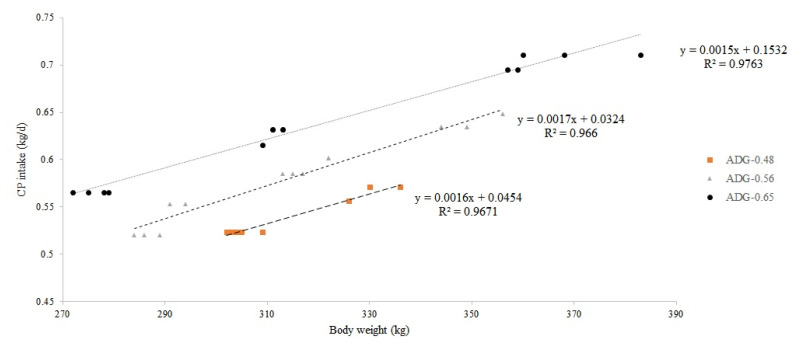
Regression equation for calculation of crude protein (CP) requirements (kg/d) of Hanwoo heifers. ADG, average daily gain. ADG-0.48, y = 0.0016x+0.0454 (R^2^ = 0.9671), ADG-0.56, y = 0.0017x+0.0324 (R^2^ = 0.9660); ADG-0.65, y = 0.0015x+0.1532 (R^2^ = 0.9763).

As a result of the experiment in this study, regression equa tions for calculation of TDN (Figure 2) and CP requirements (Figure 3) were derived according to the observed BW by the level of ADG. In addition, based on the ADG results, regression equations for TDN [Eq. 1] and the CP requirements [Eq. 2] according to the target ADG level and BW were derived as follows:


[Eq. 1] $$\text{TDN }(\text{kg}/\text{d})=0.3466+(0.0068\times \text{BW})+(0.4250\times \text{ADG})\;({\text{R}}^{2}=0.8806)$$

[Eq. 2] $$\text{CP }(\text{kg}/\text{d})=0.0665+(0.0014\times \text{BW})+(0.1105\times \text{ADG})\;({\text{R}}^{2}=0.8479)$$

Comparing results from regression equations [Eq. 1, 2] and NIAS (2012, 2017) [1,2] for TDN and CP requirements according to the target daily gain based on the 300 kg BW of heifer are presented in Table 4. When compared with NIAS (2012) [1], TDN requirements were 26.66%, 28.19%, and 29.37% lower, respectively, and CP requirements were 5.81%, 10.05%, and 13.63% lower, respectively. In addition, compared with NIAS (2017) [2], 20.27%, 23.46%, and 26.01% lower values for TDN and 4.04%, 6.89%, and 9.38% lower values for CP were derived, respectively. Compared with the Korean Feeding Standard for Hanwoo, the TDN and CP requirements derived from this experiment show that the reduction rate increases as the target daily gain increases from 0.2 to 0.6 kg/d. This is because the coefficient of both TDN and CP for ADG in the regression equation derived from this experiment is lower than that of the regression equation [TDN = 0.169+(0.009×BW)+(1.176×ADG); CP = 0.009+ (0.002×BW)+(0.119×ADG)] of NIAS [2]. It is also because the nutrient requirement value at the daily gain of 0 kg/d was lowered.

There was no significant difference in FCR between treat ment groups in the 1st period, but during the 2nd period, they were16.20, 10.57 and 8.29, respectively, with T-0.2 being higher than T-0.4 and −0.6 (p<0.05). The whole period was 11.79, 10.66, and 9.54, respectively, indicating that the lower the target daily gain, the higher the feed requirement. This is because, although there was no difference in DMI among treatment groups, T-0.2 fed a relatively high proportion of rice straw resulting in low daily gain.

### Animal behavior

The results from animal behavior according to the energy and protein levels in the feed of are shown in Table 5. The experiment was observed by dividing day time (0700 to 1800, 11 h) and night time (1800 to 0700, 13 h) throughout the day. It was confirmed that the majority of standing, eating, walking and drinking occurred during the day time, while the majority of lying and rumination occurred at the night time. In other words, standing, eating, walking, and drinking in the day time were 73%, 100%, 65%, and 94%, respectively, if the average value of the three treatments was calculated as a percentage based on the time data. Lying and rumination in night time were 86% and 76%, respectively.

Lying, standing and walking of T-0.2, −0.4, and −0.6 showed no significant difference in day time, night time and a whole day. Lying, standing, and walking are behaviors that can confirm the degree of anxiety, discomfort and comfort, and no significant difference was found in this experiment [10]. In addition, in the case of anxiety, crying is accompanied while walking [10], but such behavior was not observed during this experiment. The purpose of this study was not to focus on changes in animal behavior due to hunger stress but to examine changes in the BW of heifers while minimizing these stresses. Therefore, it was confirmed that there was no change in normal behavior, such as increased standing and walking time due to hunger stress, even if the energy and protein levels in the feed were lower than the required amount when the DMI was constant by adjusting the roughage and concentration ratio.

Eating time was longer (p <0.05) at T-0.2 than T-0.6 during the day time. The eating time becomes shorter when the amount of feed intake is low or the ratio of forage intake is low [12]. The NDF intake also affects chewing activity (eating and rumination), and is known to be from 111 to 209 minute (min) per kg of ingested NDF [13,14]. In this experiment, since the DMI was constant, it is thought that this difference appeared due to the effect of the difference in the ratio of the roughage intake and the resulting NDF intake.

The rumination affected by the ratio of roughage intake [12] also showed a significant difference (p<0.05) by the treatment groups at whole day (281.91, 331.57, and 247.28 min/24 h) and day time (65.31, 99.68, and 44.75 min/11 h). However, the longest time was observed at T-0.4, not T-0.2 where the ratio of roughage intake was highest. Since there was no significant difference in rumination at night time, the significant difference found in this behaviour is due to day time. Day time is the time of highest activity during a 24 hour period, and rumination is not the main activity. Therefore, it is considered that the significant difference may be due to the individual difference, not the feed.

Drinking is affected by the type of feed [15] and moisture content [16]. In this experiment, a significant difference between treatment groups was shown as 0.17, 0.19, and 0.61 times (p<0.05) in the number of drinking at night time, but the difference between among groups was less than 1 time, so it is unreasonable to judge the effect of feed according to energy and protein levels.

## CONCLUSION

As a result of the experiment according to the energy and protein levels in the diet of Hanwoo heifers, the treatment groups with the target daily gain of 0.2, 0.4, and 0.6 kg/d showed 0.48, 0.56, and 0.65 kg/d (p<0.05), respectively. Based on these results, a regression formula for predicting TDN and CP requirements according to ADG and BW was derived, and TDN and CP requirements were newly derived based on 300 kg heifers. Comparing the newly derived requirements with NIAS (2017)[2], TDN requirements for T-0.2, −0.4, and −0.6 were 20.27%, 23.46%, and 26.01% lower, and CP requirements were 4.04%, 6.89%, and 9.38% lower. In addition, a longer eating time was observed in T-0.2 with a high ratio of roughage intake compared to T-0.6. However, formulas for feeding standards will require numerous repeated experiments and a longer period of experiment. Furthermore, in case of Hanwoo, improvement in performance such as growth rate and meat quality is rapidly progressing due to genetic improvement through artificial insemination, feed manufacturing technology, and feeding management. Therefore, the feeding experiments on the nutrient requirements of Hanwoo heifers should be continued in the future.

## Figures and Tables

**Table 1:** Chemical composition of formula feed and rice straw, and ingredients of the formula feed

Items (%)	Formula feed	Rice straw
Chemical composition (DM, %)		
DM	88.90	81.07
CP	14.99	4.64
EE	3.24	1.37
CF	8.85	32.37
Ash	9.61	12.59
NDF	30.01	66.96
ADF	16.33	43.71
ADL	13.12	35.45
NFE	52.22	30.10
TDN	64.19	28.30
Ingredients (as-fed, %)		
Wheat	10.0	
Corn	5.0	
Wheat flour	5.0	
Corn gluten feed	15.0	
Rice polishings	2.9	
Wheat bran	11.0	
Tapioca	15.0	
Coconut milk	13.0	
Molasses	5.0	
Palm kernel meal	13.0	
Limestone	4.0	
Salt	0.8	
Vitamin and mineral premix^1)^	0.3	
Total	100.0	

DM, dry matter; CP, crude protein; EE, ether extract; CF, crude fiber; NDF, neutral detergent-insoluble fiber; ADF, acid detergent-insoluble fiber; ADL, acid detergent lignin; NFE, nitrogen-free extract; TDN, total digestible nutrients.

1) Vitamin and mineral premix provided following nutrients per kg of diet: vitamin A 9,805 IU, vitamin D_3_ 1,961 IU, vitamin E 3.89 IU, Fe 48.84 mg, Mn 16.28 mg, Zn 16.28 mg.

**Table 2:** Behavioral characteristics of Hanwoo heifers recorded by CCTV

Item	Definition
Lying	Legs and lower abdomen contact with the ground
Standing	All legs are extended and supporting the body
Eating	Standing and bowing down to the feed
Rumination	Chewing while lying and standing without feed
Walking	Moving over three footprints in a standing position
Drinking	Head over or in the water trough

**Table 3:** Effects of dietary energy and protein levels on performance of Hanwoo heifers

Item	Treatments^1)^			SEM

	T-0.2	T-0.4	T-0.6	
BW (kg)				
Initial (0 d)	316.7	318.3	320.6	8.044
Second (36 d)	337.9	339.1	343.5	9.566
Final (63 d)	348.3	353.6	361.2	9.376
DMI (kg/d)				
Formula feed	2.67	3.04	3.44	0.069
Rice straw	3.07	2.85	2.63	0.057
DMI	5.75	5.88	6.07	0.126
TDN	2.58	2.75	2.95	0.060
CP	0.54	0.59	0.64	0.013
ADG (kg/d)				
1st period	0.59	0.57	0.62	0.054
2nd period	0.36^a^	0.56^b^	0.72^c^	0.048
Whole period	0.48^a^	0.56^ab^	0.65^b^	0.038
FCR (F/G)				
1st period	9.36	10.76	9.73	0.833
2nd period	16.20^b^	10.57^a^	8.29^a^	1.507
Whole period	11.79^b^	10.66^ab^	9.54^a^	0.642

SEM, standard error of the means; BW, body weight; DMI, dry matter intake; TDN, total digestible nutrient; CP, crude protein; ADG, average daily gain; FCR, feed conversion ratio.

1) The target average daily gain was 0.2 (T-0.2) 0.4 (T-0.4), and 0.6 kg/d (T-0.6) by controlling the amount of rice straw.

a–c Means within a row in a same parameter that do not share a letter differ at p<0.05.

**Table 4:** Comparison of nutrient requirements (kg/d) for growth in 300 kg Hanwoo heifer

Items	Treatments^1)^		

	T-0.2	T-0.4	T-0.6
TDN			
NIAS (2012)^2)^	3.37	3.56	3.74
NIAS (2017)	3.10	3.34	3.57
Calculated value^3)^	2.47	2.56	2.64
CP			
NIAS (2012)	0.54	0.59	0.64
NIAS (2017)	0.53	0.57	0.61
Calculated value	0.51	0.53	0.55

TDN, total digestible nutrient; CP, crude protein; BW, body weight.

1) The target average daily gain was 0.2 (T-0.2), 0.4 (T-0.4) and 0.6 kg/d (T-0.6) by controlling the amount of rice straw.

2) National Institute of Animal Science. Korean feeding standard for Hanwoo.

3) Calculated value by the equation from this study:

$$\begin{array}{l} \text{TDN }(\text{kg}/\text{d})=0.3466+(0.0068\times \text{BW})+(0.4250\times \text{ADG})\;({\text{R}}^{2}=0.8806).\\ \text{CP }(\text{kg}/\text{d})=0.0665+(0.0014\times \text{BW})+(0.1105\times \text{ADG})\;({\text{R}}^{2}=0.8479). \end{array}$$

**Table 5:** Observation of animal behaviors (min) in Hanwoo heifers fed different energy and crude protein intake levels

Items^1)^	Lying	Total standing^2)^	Standing^3)^	Eating	Rumination^4)^	Walking	Drinking (min/frequency)
	------------------------------------------------------------------ Whole day (0000–2400, 24 h) ------------------------------------------------------------------						
T-0.2	699.60	740.40	558.02	130.91^b^	281.91^ab^	43.80	7.680/04.610
T-0.4	710.40	729.30	560.10	128.35^ab^	331.57^b^	35.05	5.800/04.130
T-0.6	706.86	733.12	564.47	116.45^a^	247.28^a^	41.60	10.630/05.110
SEM	33.646	33.646	35.141	4.980	17.164	9.590	2.145/00.617
	------------------------------------------------------------------ Day time (0700–1800, 11 h) ------------------------------------------------------------------						
T-0.2	104.31	555.69	393.86	130.90^b^	65.31^a^	25.41	7.420/0 4.44
T-0.4	90.14	569.86	415.22	128.35^ab^	99.68^b^	22.22	5.580/0 3.94
T-0.6	93.89	566.11	411.69	116.45^a^	44.75^a^	30.39	9.360/0 4.22
SEM	21.670	21.670	22.009	4.980	12.094	4.171	2.168/0 0.523
	------------------------------------------------------------------ Night time (1800–0700, 13 h) -------------------------------------------------------------------						
T-0.2	595.29	184.71	164.16	-	216.61	18.39	0.260/0 0.17^a^
T-0.4	620.56	159.44	144.86	-	231.88	12.83	0.230/0 0.19^a^
T-0.6	612.97	167.03	152.78	-	202.53	11.21	1.270/00.61^b^
SEM	18.080	18.080	20.443	-	10.407	9.300	0.445/0 0.130

SEM, standard error of the means.

1) The target average daily gain was 0.2 (T-0.2) 0.4 (T-0.4) and 0.6 kg/d (T-0.6) by controlling the amount of rice straw.

2) Include eating, rumination, walking and drinking.

3) Exclude eating, walking and drinking.

4) Included in both lying and standing.

a,b Means within a column in a same parameter that do not share a letter differ at p<0.05.
